# Positive fluid balance as a risk factor for mortality and acute kidney injury in vasoplegic shock after cardiac surgery

**DOI:** 10.1186/cc14271

**Published:** 2015-03-16

**Authors:** A Rezende, L Camara, A Leme, J Ribeiro, I Bispo, S Zeferino, J Jardim, C Park, E Osawa, J Almeida, A Gerent, F Galas, D Fonseca, J Fukushima, L Hajjar

**Affiliations:** 1 ICESP, São Paulo, Brazil; 2 Heart Institute, University of São Paulo, Brazil

## Introduction

After cardiac surgery, about 15% of patients develop vasoplegic shock, characterized by systemic vasodilation, increased capillary permeability and edema. We hypothesized that large-volume resuscitation, resulting in positive fluid balances in the first 24 hours of ICU admission, would be associated with mortality and would not be protective against AKI in this subset of patients.

## Methods

This is a retrospective analysis of 362 patients submitted to cardiac surgery at the Heart Institute of University of São Paulo in a period of 2 years. Of a total of 2,383 patients, we enrolled 362 patients. Vasoplegic shock was diagnosed if in the 24 hours of ICU admission patients had hypotension, need of vasoactive drugs after fluid replacement and cardiac index ≥2.2 l/minute/m^2^. Data were analyzed in logistic regression models for 30-day mortality and acute kidney injury through Acute Kidney Injury Network (AKIN) score as outcomes.

## Results

The mean age of patients was 57 years. Of 362 patients, 53 died at 30 days (14.6%). Nonsurvivors as compared with survivors were slightly older (59 ± 12 vs. 55 ± 13, *P *= 0.063), had a higher prevalence of AKI through AKIN score ((0) 6.9%, (1) 11.1%, (2) 28.9%, (3) 31.9%, *P *< 0.001), a higher 24-hour fluid balance (421 ml (-55 to 695) vs. 2,686 ml (1,321 to 2,856), *P *< 0.001), and higher lactate levels at the intraoperative and at 48 hours (5 mmol/l (4.0 to 7.6) vs. 4.4 (3.33 to 6.55), *P *< 0.001; and 8.11 (5.49 to 12.3) vs. 1.5 (1.33 to 1.88), *P *< 0.001). In the multivariate analysis, positive fluid balance in the first 24 hours (OR = 1.006, 95% CI = 1.003 to 1.008, *P *< 0.001) and higher lactate after 48 hours (OR = 1.204, 95% CI = 1.072 to 1.353, *P *= 0.002) were predictors of 30-day mortality. Forty-three percent of patients developed AKI during 30 days. In the multivariate analysis, positive fluid balance in the first 24 hours (OR = 1.001, 95% CI = 1.000 to1.001, *P *< 0.001) and higher lactate at 48 hours (OR = 1.011, 95% CI = 1.000 to 1.021, *P *= 0.0043) were predictors of 30-day AKI.

## Conclusion

Positive fluid balance after cardiac surgery is an independent risk factor for mortality and for acute kidney injury in patients presenting vasoplegic shock.

